# A Case Report of Complete Resolution of Auricular Mucormycosis in an 18-Month-Old Diabetic Child

**DOI:** 10.1155/2021/6618191

**Published:** 2021-02-20

**Authors:** Mariam Aljehani, Hatem Alahmadi, Mansour Alshamani

**Affiliations:** ^1^Department of Otolaryngology, Ohud hospital, Al Madinah Almunwarah, Madinah, Saudi Arabia; ^2^Department of Pediatric Infectious Disease, Maternity and Children Hospital, Al Madinah Almunwarah, Madinah, Saudi Arabia; ^3^Department of Otology, Neurotology and Cochlear Implant Surgery, Program Director of the Saudi Board of Otolaryngology, Ohud Hospital, Al Madinah Almunawarah, Madinah, Saudi Arabia

## Abstract

**Background:**

One of the most rare but deadly types of infectious fungal infection is Mucormycosis. All the cases reported with this type of infection are immunocompromised individuals. The challenge of early detection and intervention makes it one of the high mortality rates among other infectious diseases. *Case Report*. We report an 18-month-old girl with undiagnosed diabetes presented with a very aggressive form of necrotic infection of the ear auricle with facial nerve palsy. Using a series of magnetic resonance imaging, antibiotics, and high clinical suspicion, a diagnosis was established, and the patient was sent to the operation theatre for surgical debridement. Monthly follow-ups showed improvement of the facial palsy, and a plan for artificial auricle is set to occur in the following months before the age of five. *Discussion*. Mucormycosis is considered a very fatal and aggressive infection that has a very high mortality rate in immunocompromised patients. Early detection of such cases with an array of magnetic resonance imaging (MRI) and computed tomography (CT) is crucial in early treatment. Early aggressive surgical debridement and empirical coverage of bacterial, viral, and fungal infections can also alleviate the chances of preventing any secondary infection to develop in such cases.

**Conclusion:**

A combination of antifungal, antibiotic, and antiviral with timely surgical intervention improved the patient with complete resolution of the facial nerve palsy and no further recurrence of the infection.

## 1. Introduction

Mucormycosis is a type of fungal infection, which occurs mostly in diabetic patients, belonging to the order *Mucorales* [1]. It is the immunocompromised status of diabetic individuals that makes them more susceptible to such an aggressive infection as they can be prone to impaired phagocytic function which may occur during the ketoacidosis state [1, 2]. The fungal spore's inoculation that deposit in the cutaneous part of the body is hypothesized to be often occurring from the result of injury, while some report it as an air-borne type of transmission pathogen [2, 3].

Mucormycosis infection is usually found predominantly in paranasal sinuses, while other places such as the gastrointestinal tract or cutaneous areas of the body are very rarely reported [3]. Due to the aggressiveness of the fungi, once the infection appears in the external auditory canal or the pinna, it is classified as a malignant otitis externa (MOE) until proven otherwise [4]. Such cases require urgent care, early diagnosis, and time-efficient treatment as it can be life-threatening among immunocompromised patients. However, its incident in the pediatric age group is very rare with a high mortality rate [5]. Therefore, the reporting of such cases will broaden the mindfulness while approaching diabetic children who are susceptible to such aggressive fungal infections.

## 2. Case Report

**Figure 1:**
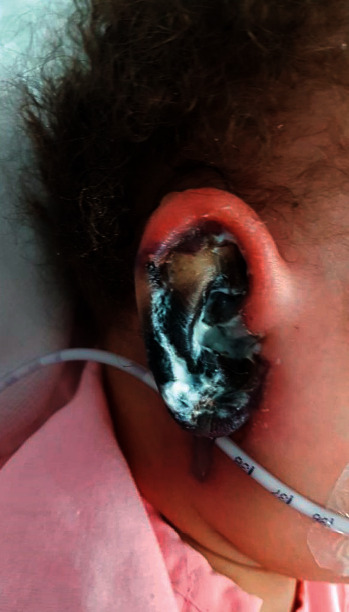
The patient's ear upon presentation.

An 18-month-old girl was admitted to the hospital in the pediatric endocrinology department with a newly discovered diagnosis of diabetes mellitus (DM) in the form of diabetic ketoacidosis (DKA). Initially, the patient presented three weeks before admission to the emergency department by her parents with a history of skin rash in upper and lower limb which had a vesicle-like appearance. Upon further questioning, the parents stated that she was not vaccinated and reported a history of decreased activity, generalized fatigue, and persistent weight loss for three weeks, and after proper evaluation, she was diagnosed as a case of chickenpox and was discharged at the same day after providing supportive treatment. One week later, she returned to the emergency department after developing a persistent fever of 38°C, vomiting, abdominal pain, right ear discoloration for three days, which was bluish to dark in color, followed by discharge two days later, and an ear swelling (Figure 1). After further evaluation, the patient was diagnosed as a case of DM and was admitted to the pediatric intensive care unit as a case of DKA for further stabilization. After stabilizing the patient, her ear discoloration and swelling were increasing rapidly. The otolaryngology team was involved, and a multidisciplinary approach was established moving forward. Regarding the initial assessment, the ear appeared swollen, with necrotic-like discoloration, discharge was oozing, and aright-side facial nerve palsy was reported. Furthermore, there was no skin lesion or rash noted, no history of a recent surgical procedure or trauma, no previous history of meningitis or congenital malformation at birth, and no family history of similar conditions.

Regarding her physical examination, a right wet gangrenous black-to-brownish discoloration of the auricle was noted. In addition, an edematous external auditory canal with seminarrow external opining was noted, while a vesicle on the upper helix of the cartilage was seen. The discoloration was mainly found on the auricle and extended to the tail of parotid involving the right side of the face with a House–Brackmann (HB) score of grade IV of the facial nerve. Upon palpation, there was no palpable cartilage felt, and the swelling was extended to the neck towards the upper cervical area measuring around 1 × 2 cm in size. The swelling was firm, hard, red, and tender.

Regarding her lab investigation, a complete blood count was normal except for lymphopenia and persistent decreasing eosinophilia throughout her admission. Meanwhile, her arterial blood gas showed an acidotic picture consistent with her DKA, and her renal and liver function tests were normal. While a decision to further stabilize her DKA status, a right ear swap was taken, and a fungal organism was identified by the laboratory as the causative pathogen which was Mucormycetes. Similarly, blood and urine culture showed a similar result of Mucormycosis. Keeping in mind that the patient presented during the pandemic crisis of COVID-19 at that time of the incident, a nasopharyngeal swap was negative. Further evaluation using computed tomography (CT) and magnetic resonance image (MRI) were used to determine the spread of the disease and evaluate possible extensive bony and ear involvement.

**Figure 2:**
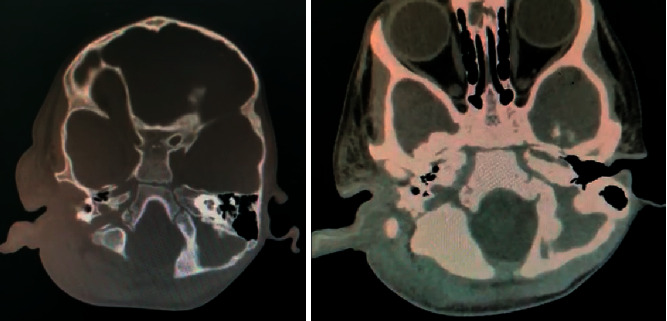
CT scan showing diffuse left side periauricular swelling.

**Figure 3:**
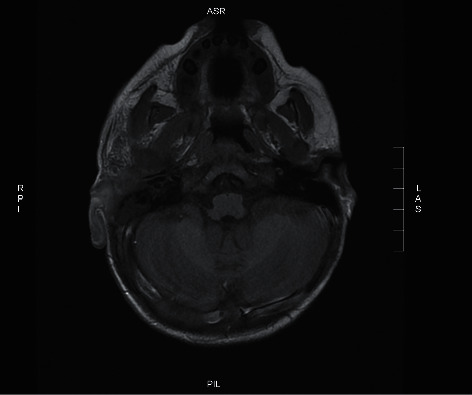
T2 MRI with no contrast showing significant inflammatory changes in the right ear canal, periauricular region.

**Figure 4:**
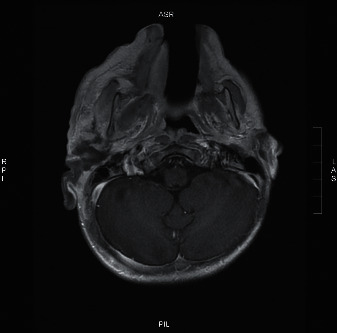
T1 MRI with gadolinium contrast showing significant inflammatory changes in the right ear canal, periauricular region.

**Figure 5:**
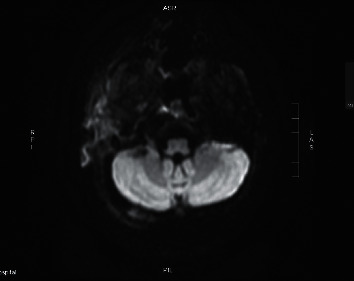
Diffusion-weighted imaging showing significant inflammatory changes in the right ear canal, periauricular region.

The CT scan showed a heterogenous density, diffuse enhancement, periauricular soft tissue swelling, and fat stranding of the right ear (Figure 2). Afterwards, a number of MRI sequences were utilized to visualize the lesion. It included MRI with no contrast (Figure 3), MRI using gadolinium-contrast media (Figure 4), and diffusion-weighted imaging (DWI) (Figure 5). The MRI sequences found significant inflammatory changes in the right ear canal, periauricular region, masticator, and parotid spaces with obliteration of fat plane between the parotid gland and masticator space. In addition, there was periauricular ill-defined collection found extending antero-inferior to parotid space using high T2 signal intensity and low T1 signal intensity with no restriction and no enhancement in postcontrast images. Lastly, fluid signal intensity was noted with deformed right middle ear cavity and mastoid air cells associated with and destroyed the entire right mastoid bone, and the DWI confirmed our suspicions of mucormycosis excluding malignant otitis externa.

**Figure 6:**
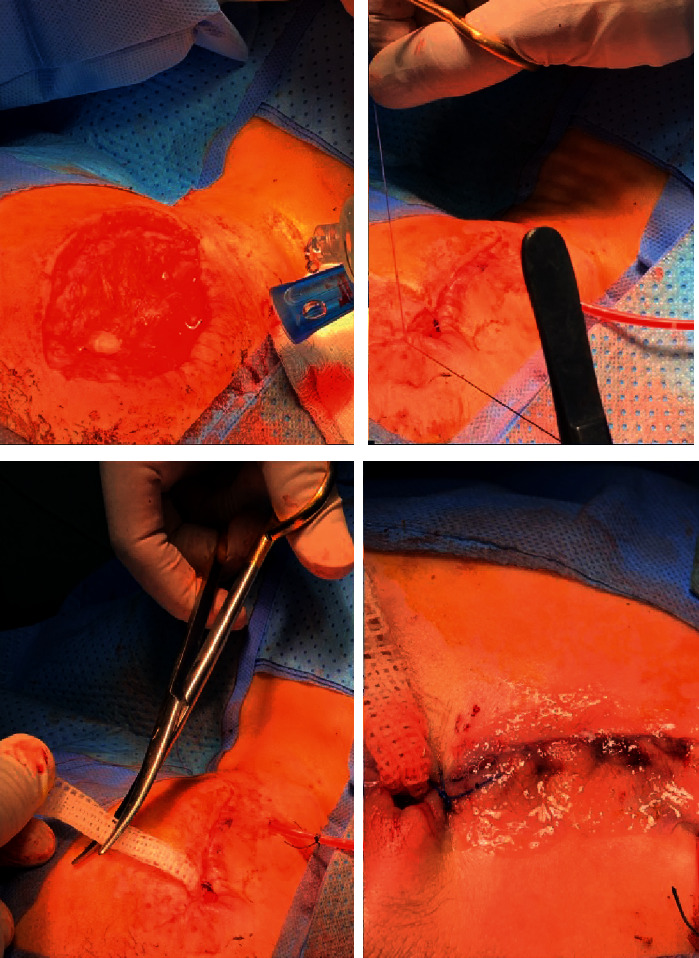
Intraoperative wound closure with pack and drains inserted.

**Figure 7:**
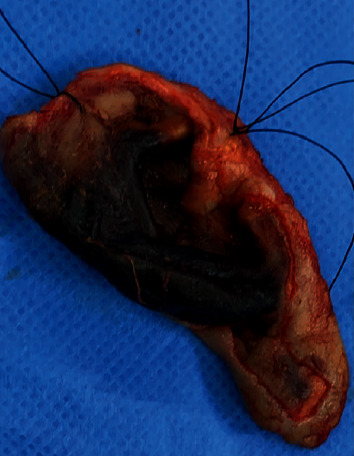
Necrotic right ear auricle after excision.

**Figure 8:**
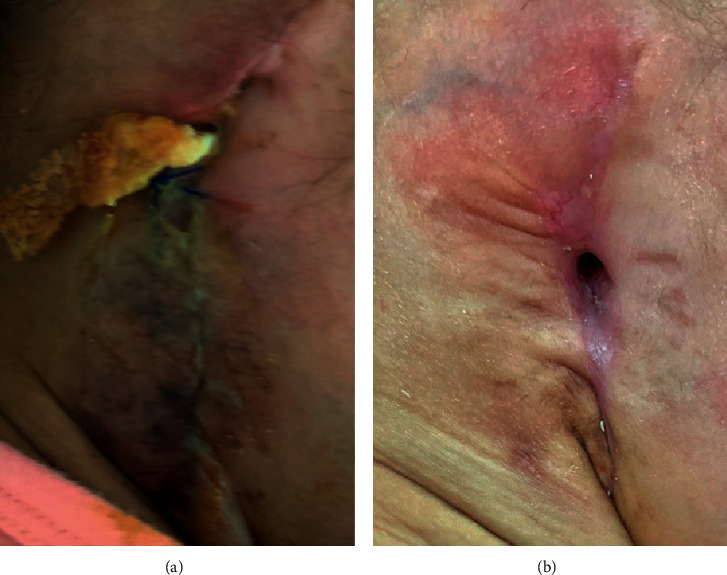
(a) Day 2 postoperatively showing packing of the ear and suture. (b) Day 45 postoperatively, showing complete healing of the wound with no discharge or inflammatory signs.

**Figure 9:**
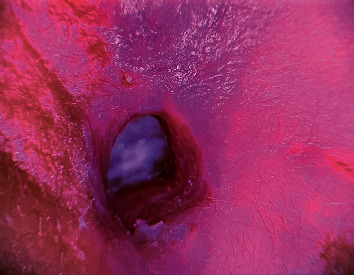
Day 45 view of the right external auditory canal with an intact tympanic membrane.

As a result, the patient was operated on as a life-saving measurement. We did an extensive surgical debridement including total excision of the auricle and cartilaginous part of the external auditory canal. The incision was extended to involve the upper part of the neck to excise the involved necrotized skin, subcutaneous tissue, lymph nodes, and the upper anterior part of the sternocleidomastoid muscle. The excision was done with a safe margin of around 5–7 mm, and then primary closure of the surgical defect with preservation of the patency of external auditory canal was done (Figure 6). No middle ear surgery was performed as there were no signs of involvement clinically to support the radiological finding. The excised ear (Figure 7) was sent for histopathology and reported extensive necrosis with acute on top of chronic inflammation, heavy infiltration of neutrophils, and particles of mucormycosis-like were seen. The patient was sent to the PICU for close observation of the drain and daily dressing. On the second postoperative day, she was extubated, drain was removed, and ear drops and sterile packs were inserted to keep the external auditory canal patent (Figure 8). Regarding her medication, dexamethasone, vancomycin, acyclovir, linezolid, meropenem, and amphotericin B with drug-level monitoring were started postoperatively. One week later, she was shifted to the normal pediatric ward with noted improvements, stable vital signs, and better overall general condition. The patient was then discharged on the same antibiotics, and a follow-up appointment for MRI was set 6 weeks after discharge. The follow-up MRI, microscopic ear examination, and numerous aural swabs and blood cultures revealed no definitive recurrence of mucormycosis, and the wound healed very well with an intact tympanic membrane (Figure 9). Monthly follow-ups showed improvement of the facial palsy to HB grade III, and a plan for artificial auricle is set to occur in the following months before the age of five.

## 3. Discussion

In 1885, Paltauf was the first one to describe mucormycosis and the disease associated with it by a saprophytic fungus, mainly *Mucor* or *Rhizopus*. Since that time, mucormycosis is considered the deadliest and the most rapidly progressive form of fungal infection found in humans [6]. *Rhizopus* organisms have an enzyme, ketone reductase, which allows them to thrive in high glucose, acidic conditions. Serum from healthy individuals inhibits the growth of *Rhizopus*, whereas serum from individuals in diabetic ketoacidosis stimulates growth, thus making diabetics more prone to infection [7, 8]. Moreover, children with malignant external otitis have a higher incidence of facial palsy due to their relatively undeveloped mastoid process and the more medial location of the fissures of Santorini, which places the facial nerve in closer proximity to the ear canal.

In order to differentiate between malignant otitis externa, cholesteatoma, and other forms of necrotizing fasciitis, a number of contrast MRI and DWI should be performed as these cases are time-sensitive and should be tackled the correct way with an accurate diagnosis as much as possible. The role of DWI has been established by Geneidi EAS et al. in differentiating otitis media and cholesteatoma in which DWI showed very good sensitivity and excellent specificity in the detection of diffusion restriction of cholesteatoma compared to chronic otitis media which appears as a facilitated diffusion [9]. Furthermore, Gallium scans has been found to be more specific in detecting infection due to their ability to incorporate within granulocytes as well as the bacteria, giving it more superiority in that matter when compared to other modalities [10]. In our case, utilizing both DWI and gadolinium contrast gave us more confidence in excluding malignant otitis externa as the culprit.

Kermani et al. described in his retrospective study four cases of mucormycosis. The first case was a 3-year-old girl with poor overall health condition who had the infection as a picture of mastoiditis. It is worth noting that she developed an anaphylactic shock while on amphotericin B unlike our patient who tolerated the drug extremely well. Furthermore, she was operated on but died four days later due to complication of sepsis, lateral sinus thrombosis, and multiorgan failure [3]. Compared to our patient, she had a very good postoperative course and was better with the combination therapy that we initiated and monitored closely with careful tittering. Kermani et al., in his second case, describe an 18-year-old male with type 1 DM. The patient with mucormycosis was found in his maxillary sinus, hard palate, and inflammation of the periorbital region as a picture of cellulitis. The patient was also started on amphotericin B with surgical curettage of the palatine lesion. His symptoms resolved after 9 months completely except persistent grade II facial palsy. Compared to our patient, the infection was found in the ear auricle where her facial palsy was completely resolved 2 months later. Lastly, the last two cases were in relatively older patients who were also type 1 DM of 24- and 77-year-old males. They had a similar course with amphotericin B, and their symptoms resolved completely in 10 months [3].

Bellazreg F et al. described five cases of mucormycosis which were 4 male and 1 female. The outcome was unfavorable in 3 of these patients with death marking a mortality rate of their cases of 60%. The youngest of them was a 25-year-old male with type 2 DM, while the oldest was a 77-year-old male with no immunocompromised conditions. Only two of them had surgery, a 25 year-old male, who died due to multiorgan failure, and a 72 year-old-female, who had complete resolution. Even though our patient is very young compared to these cases, it is important to compare all cases of mucormycosis due to its rare incident in the literature. Bellazreg F et al. used amphotericin B solely in treatment of the fungi. However, in our patient, there was a slight delay in the surgical intervention of the case due to her DKA status which required stabilization before proceeding to operation [11]. The combination of amphotericin B, meropenem, dexamethasone, linezolid, vancomycin, and acyclovir can be argued to provide a better edge in attacking concurrent undetectable organisms, therefore increasing the likelihood of survivability and resolution.

Previous papers reporting this type of infection are very limited to young age. The only reported mucormycosis of young patients either died or had persistent unresolved facial palsy [3, 12]. Cranial nerve affection is commonly found in those types of fungal infections in that region [13]. Therefore, careful monitoring and examination is needed as it might play a role in determining the outcome. Regarding surgical approach, a complete debridement of all necrotic lesions with healthy margins should be done. Our safe margins of healthy tissues were determined around 5–7 mm which showed great outcome in our postoperative monitoring and resolution with no recurrence one year later.

This is the first case of mucormycosis of a child that completely resolved with marked improvements of the facial palsy. Furthermore, the rest of the papers reporting mucormycosis in adults were mostly immunocompromised with diabetes and a high mortality rate regardless of proceeding to surgery or not [2, 3, 11, 14]. Nonetheless, all cases used a single therapy of amphotericin B, while we started a multidimensional therapy on the hypothesis of availability of a secondary infection due to the rapid progressiveness of the necrosis. Continuous monthly MRI monitoring using DWI as well as MRI with gadolinium contrast can prove beneficial with many studies supporting it in the literature [10, 11].

More studies should be targeted moving forward on the use of DWI in combination of gadolinium contrast MRI in detecting mucormycosis. Furthermore, the multiorganismal dimensional therapy should be utilized based on the hypothesis of having such immunocompromised patients who can be under other infections that are not apparent due to the severity of mucormycosis, therefore helping the body in attacking this aggressive infection and avoiding multiorgan failures.

## 4. Conclusion

Mucormycosis infection is quite rare in the head and neck region, mainly in the ear. It is often found in immunocompromised patients with acute progression of necrotizing form and high mortality rate. Early diagnosis coupled with aggressive medical and surgical approach should give those patients better survivability chance. MRI DWI and gadolinium contrast can prove specificity in detecting and monitoring mucormycosis of the auricle and external auditory canal. The combination of amphotericin B, meropenem, dexamethasone, linezolid, vancomycin, and acyclovir can also be used as our patient had complete resolution of the infection postoperatively given the importance of establishing early diagnosis and timely surgical intervention to avoid a high mortality rate of such cases.

## Data Availability

The authors declare that data supporting the findings of the study are available within the article.
