# Corrigendum to “A Case Report of Complete Resolution of Auricular Mucormycosis in an 18-Month-Old Diabetic Child”

**DOI:** 10.1155/2021/9794624

**Published:** 2021-04-17

**Authors:** Mariam Aljehani, Hatem Alahmadi, Mansour Alshamani

**Affiliations:** ^1^Department of Otolaryngology, Ohud Hospital, Al Madinah Almunwarah, Madinah, Saudi Arabia; ^2^Department of Pediatric Infectious Disease, Maternity and Children Hospital, Al Madinah Almunwarah, Madinah, Saudi Arabia; ^3^Department of Otology, Neurotology and Cochlear Implant Surgery, Program Director of the Saudi Board of Otolaryngology, Ohud Hospital, Al Madinah Almunwarah, Madinah, Saudi Arabia

In the article titled “A Case Report of Complete Resolution of Auricular Mucormycosis in an 18-Month-Old Diabetic Child” [1], the authors would like to correct the figure legend of Figure 2 from:

“Figure 2. CT scan showing diffuse left side periauricular swelling.”to

“Figure 2. CT scan showing diffuse right side periauricular swelling.”

The authors would additionally like to clarify that written informed consent was obtained from the parents for the publication of this case report.

The authors confirm that this does not affect the results and conclusions of the article.
